# Peri-prosthetic bone remodeling of hydroxyapatite-coated compaction short stem was not affected by stem alignment

**DOI:** 10.1186/s13018-022-03022-7

**Published:** 2022-03-03

**Authors:** Shinya Hayashi, Yuichi Kuroda, Naoki Nakano, Tomoyuki Matsumoto, Tomoyuki Kamenaga, Toshihisa Maeda, Takahiro Niikura, Ryosuke Kuroda

**Affiliations:** Department of Orthopedic Surgery, Kobe University Graduate School of Medicine, 7-5-1 Kusunoki-cho, Chuo-ku, Kobe, 650-0017 Japan

**Keywords:** Total hip arthroplasty, Short tapered-wedge stem, Full hydroxyapatite-coated compaction short stem, Bone mineral density, Stem alignment

## Abstract

**Background:**

To improve implant survival through accelerated early bone remodeling during total hip arthroplasty (THA), hydroxyapatite (HA) is widely used as a bioactive coating, which is believed to enhance initial fixation by osseointegration. We aimed to investigate the relationship between stem insertion alignment and postoperative bone mineral density (BMD) changes in patients with full hydroxyapatite-coated (HA) compaction short stem and short tapered-wedge stem.

**Methods:**

This retrospective cohort study enrolled 115 consecutive patients (115 joints) undergoing THA using the full HA compaction short (*n* = 59) and short tapered-wedge (*n* = 56) stems. Stem alignment, including anteversion, valgus, and anterior tilt were measured by a three-dimensional template using computed tomography data. Post-operative peri-prosthetic BMD was measured by dual-energy X-ray absorptiometry. The relationship between stem alignment and BMD changes in the stems was analyzed.

**Results:**

Patterns of peri-prosthetic BMD changes were similar in both groups. Stem insertion alignments of anteversion, valgus, and anterior tilt were different between the two stem types. Stem alignment of valgus and anterior tilt did not affect peri-prosthetic BMD in either of the stem type. An absolute anteversion difference between stem anteversion and original canal anteversion caused significant peri-prosthetic BMD loss in Gruen zones one and seven in the tapered-wedge stem. However, stem alignment of absolute anteversion difference did not affect BMD changes in the HA compaction stem.

**Conclusions:**

Peri-prosthetic bone remodeling remained unaffected by stem alignment after THA with the new short full HA compaction stem.

## Background

Hydroxyapatite (HA) is widely used in total hip arthroplasty (THA) to improve implant survival through accelerated early bone remodeling [1, 2]. This bioactive coating is believed to enhance initial fixation by osseointegration [1, 2]. The ACTIS stem (DePuy Synthes, Warsaw, IN, USA) is a medial-collared, triple-tapered short stem with full HA-coating, and the cancellous bone was compacted without the implant in contact with the femoral cortex. The Tri-Lock bone preservation stem (BPS) (DePuy Synthes) is a short tapered-wedge stem, which potentially preserves more bone stock, improving proximal load transfer and demonstrating lower stress shielding than long stems [3, 4]. The full HA-coated compaction short stem and short tapered-wedge stem were developed for minimally invasive THA and made from the same titanium alloy, but the geometry, type of surface coating, and concept of both stems differ substantially.

Recently, we reported that peri-prosthetic BMD changes was similar in full HA-coated compaction short stems and short tapered-wedge stems [5]. We further demonstrated that age, body mass index (BMI), and daily activity did not affect proximal femoral BMD changes in both stem types, and femoral bone shape affected the BMD changes in the tapered-wedge stem but not in the full HA compaction stem [5]. The contact force is dependent on subject-specific geometry and that specificity influences stress distribution in the peri-prosthetic bone after THA [6, 7]. A finite element method study reported that stress distribution in periprosthetic bone was dependent on stem alignment, especially anterior–posterior and varus–valgus alignments [8]. A radiographic study reported that varus alignment caused early stem loosening because of poor seating of the femoral prosthesis with decreased bone ingrowth [9]. The relation between varus–valgus alignment and stress distribution in the periprosthetic bone has been established well in biomechanical studies [9–13]. We have reported that excessive anteversion mismatch between anatomical canal and stem anteversions caused postoperative peri-prosthetic proximal BMD loss in a short tapered-wedge stem [14]. Thus, stress distribution in the peri-prosthetic bone was influenced not only by subject-specific geometry but also by stem alignment.

We hypothesized that stem alignment may affect different patterns of BMD change after THA between HA-coated compaction short stems and short tapered-wedge stems. Therefore, in this study we evaluated the relationship between stem insertion alignment and postoperative BMD change in the full HA-coated compaction short stem and short tapered-wedge stem.

## Methods

### Patients’ characteristics

**Fig. 1:**
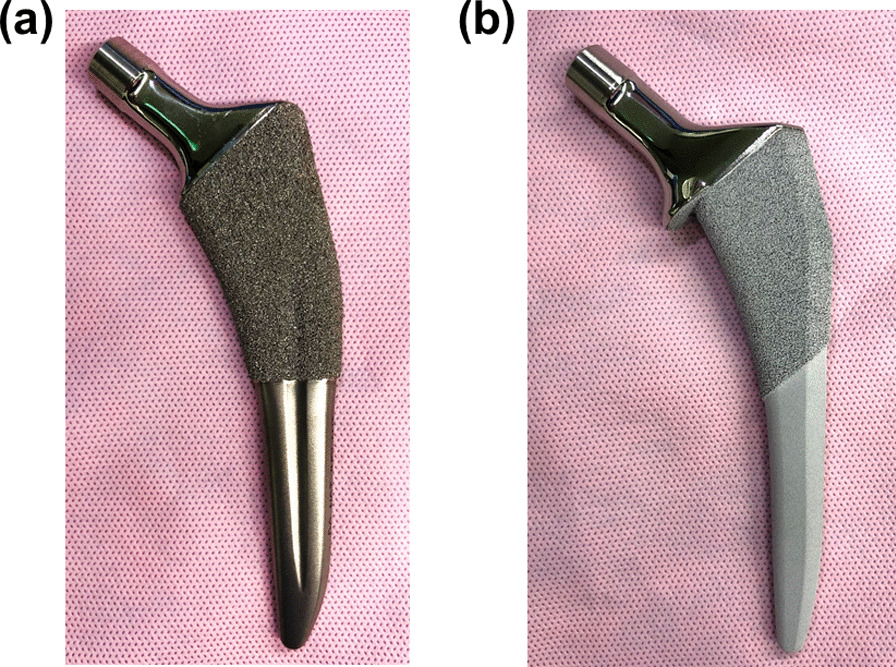
Images of **a** the short tapered-wedge stem (Tri-Lock BPS) and **b** the full HA compaction short stem (ACTIS)

A total of 159 consecutive patients (159 joints) at our institution underwent THA using the short tapered-wedge stem (Tri-Lock BPS; 95 joints) from January 2016 to April 2017 or the full HA compaction short stem from April 2017 to November 2018. To analyze the three-dimensional stem alignment, postoperative computed tomography (CT) was performed from June 2016 to July 2018. Two methods of THA were used at our institution; from June 2016 to April 2017, THA was conducted using the short tapered-wedge stem (Tri-Lock BPS; 95 joints). Thereafter, the full HA compaction short stem was introduced in Japan and, from then onward, was used at our institution. Hence, between June 2016 and April 2017, patients enrolled in the present study underwent THA using the short tapered-wedge stem (Tri-Lock BPS; DePuy Synthes; 56 joints) (Fig. 1a); then, from April 2017 to July 2018, patients underwent the full HA compaction short stem (ACTIS; DePuy Synthes, Warsaw, IN; 59 joints) (Fig. 1b). Therefore, this retrospective cohort study enrolled 115 consecutive patients (115 joints) from June 2016 to July 2018.

The preoperative diagnoses were osteoarthritis (grade 4, according to the Tönnis classification) (89 joints), avascular necrosis of the femoral head (22 joints), and rheumatoid arthritis (4 joints). Patients with a distorted anatomy of the proximal femur, osteoporosis (lumbar spine BMD < 0.8), metabolic bone disease, and those who underwent bilateral THA were excluded.

All procedures were performed via the mini-anterolateral supine approach by a single senior surgeon. Full weight-bearing was allowed for all the patients, a day after the operation. At the time of surgery, BMI was assessed. The postoperative follow-up included dual-energy X-ray absorptiometry (DEXA) scanning and evaluation of clinical factors, including hip function, which was evaluated using two grading methods: (a) the Japanese Orthopedic Association (JOA) score, which allocates 40 points for pain, 20 points for range of motion, 20 points for walking ability, and 20 points for activities of daily living, with a maximum total score of 100 points [15] and (b) the University of California Los Angeles (UCLA) activity score, which describes subjects’ level of activity from 1 (“no physical activity, dependent on others”) to 10 (“regular participation in impact sports”). The JOA and UCLA scores and radiographic findings were evaluated two years postoperatively.

### Measurement of stem alignment

**Fig. 2:**
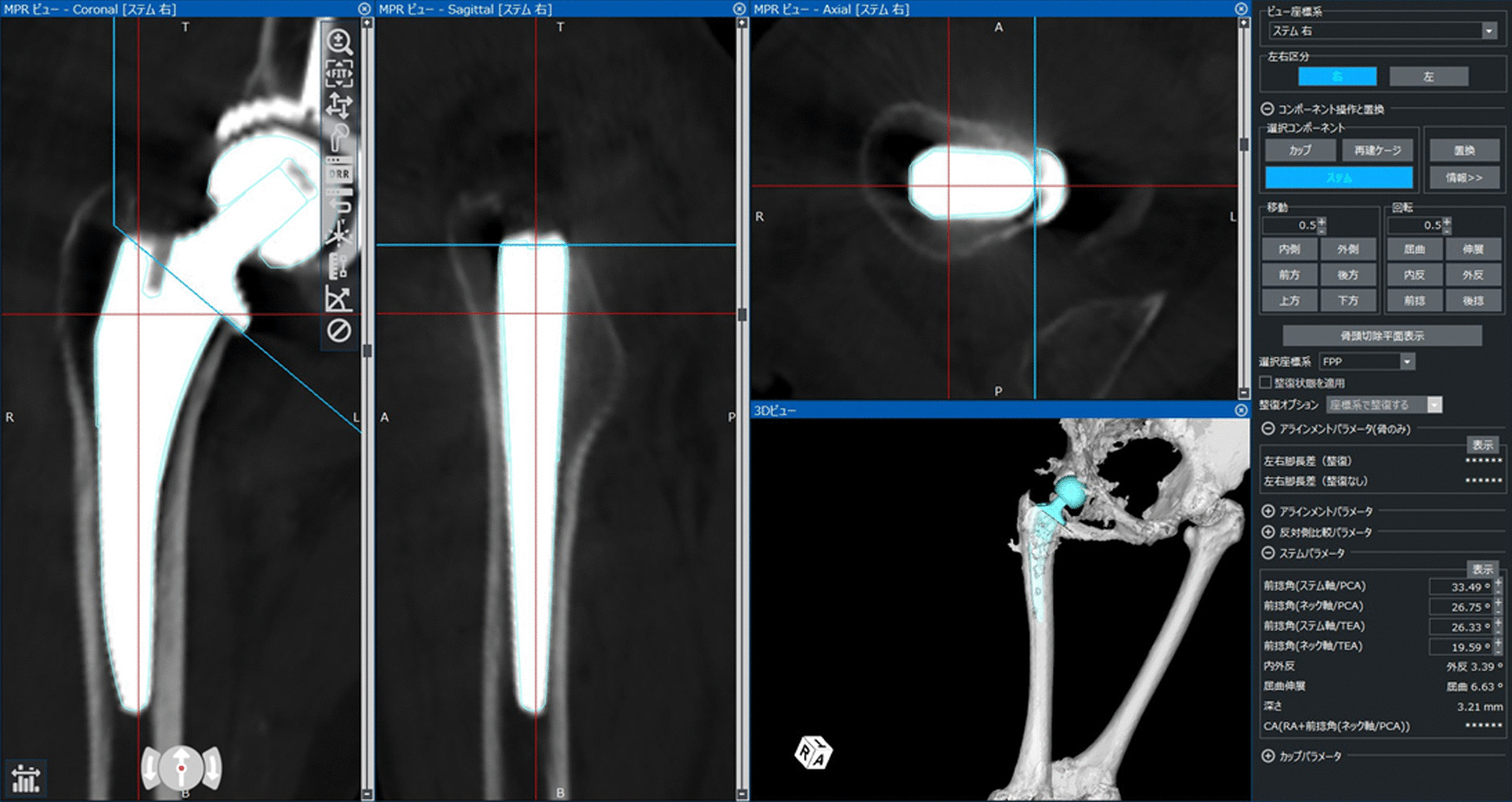
Stem alignments are measured by superimposing the templates of the stem data on postoperative images of the femoral component using Zed Hip software

Pre-operative and postoperative CT scans from the pelvis to the knee joint were performed and transferred to a three-dimensional template software (Zed Hip; Lexi, Tokyo, Japan). Computer-aided design models of the implants were manually adjusted for postoperative multiplanar reconstruction in the CT images (Fig. 2). Stem anteversion and anatomical canal anteversion angles were measured with respect to the femur’s posterior condylar line axis [16]. We compared the anatomical canal and postoperative stem anteversions, and the anteversion error was defined as the difference between the stem and anatomical canal anteversions. Patients were divided into outlier and non-outlier of stem alignment groups. The outliers of stem anteversion were defined as having an absolute difference of 5° or more between anatomical canal and postoperative stem anteversions, and the outliers of varus–valgus and anterior–posterior tilt angle were defined as having an absolute difference of 3° or more, according to previous studies [9, 14].

### DEXA measurements

A DPX-L scanner (GE Lunar Corporation, Madison, WI, USA) was used for DEXA scanning. Software version 1.35 was used for DPX-L total body scans, which set the appropriate transverse speed at 16 cm/s, 8 cm/s, or 4 cm/s, depending on the participant’s height. The patients were positioned supine with their legs in the neutral position with a knee and foot support to facilitate the scanning of the anterior–posterior projection of the proximal femur, including the area distal to the prosthesis, using an edge-detection technique.

Peri-prosthetic BMD was determined postoperatively in seven regions of interest (ROIs) based on the Gruen zones [17]. The seven Gruen zones were positioned based on the distal tip and shoulder of the prosthesis. The two most proximal Gruen zones (1 and 7) were also combined to create a proximal femur ROI, which represents the region where the porous coating of the uncemented stems is normally situated. The values were expressed as area BMD in grams per square centimeter. The BMD around the stem was assessed within 1 month (baseline BMD) and 6, 12, 18, and 24 months postoperatively by a technician in the radiology department who was blinded to the stem used. The BMD ratios were calculated by dividing each BMD value at 6, 12, 18, and 24 months postoperatively by the baseline BMD.

### Statistical analysis

All data are expressed as mean ± standard deviation (SD) unless otherwise indicated. The differences in patients’ backgrounds and stem alignment between the full HA compaction and tapered-wedge groups were analyzed using the Mann–Whitney U test (Table 1). Sequential changes in the BMD within each group were analyzed by one-way analysis of variance using the Tukey’s post-hoc test. Additionally, the BMD at each timepoint was compared between the groups using the Mann–Whitney U test (Fig. 2). The differences in peri-prosthetic BMD changes for stem valgus, anterior tilt, and absolute anteversion error alignment between outliers and non-outliers were analyzed using the Mann–Whitney U test (Tables 2, 3, 4). The difference in stem alignment and whether stem collar is contacting the femoral calcar or not were analyzed using the Mann–Whitney U test (Table 5). The differences in stem alignments among Dorr types were analyzed using the one-way analysis of variance and the Tukey’s post-hoc test (Table 6). Statistical significance was set at *p* < 0.05.

**Table 1:** Stem alignment

	Full HA compaction	Tapered wedge	*p* value
Stem anteversion	33.7 ± 11.6	32.1 ± 10.5	0.538
Valgus	3.1 ± 2.2	1.8 ± 1.8	< 0.001
Anterior tilt	6.0 ± 2.2	3.1 ± 2.1	< 0.001
Canal anteversion	33.3 ± 12.8	29.1 ± 11.1	0.101
Anteversion error	0.4 ± 7.1	3.0 ± 10.5	< 0.001
Absolute anteversion error	5.2 ± 4.8	8.8 ± 9.1	0.039

**Table 2:** Relation between stem valgus alignment and BMD change at 24 months post-operatively in the Gruen zones

	Outlier versus non-outlier (valgus alignment)									
	HA full compaction					Tapered wedge				
	Outlier (*n* = 27)	Non-outlier (*n* = 32)	*p* value	Effect size (Cohen’s d)	95% CI	Outlier (*n* = 9)	Non-outlier (*n* = 47)	*p* value	Effect size (Cohen’s d)	95% CI
Zone1	0.90 ± 0.17	0.92 ± 0.09	0.141	0.15	− 0.36 to 0.66	0.92 ± 0.12	0.90 ± 0.14	0.315	− 0.15	− 0.86 to 0.57
Zone2	0.92 ± 0.14	0.90 ± 0.09	0.159	− 0.17	− 0.69 to 0.34	0.90 ± 0.18	1.00 ± 0.10	0.014*	0.87	0.14–1.60
Zone3	1.02 ± 0.07	1.00 ± 0.03	0.084	− 0.38	− 0.9 to 0.134	1.01 ± 0.13	1.02 ± 0.18	0.805	0.06	− 0.66 to 0.77
Zone4	1.04 ± 0.07	1.04 ± 0.04	0.382	0.00	− 0.51 to 0.51	1.05 ± 0.06	1.01 ± 0.05	0.054	− 0.78	− 1.5 to − 0.05
Zone5	1.05 ± 0.10	1.03 ± 0.03	0.505	− 0.28	− 0.80 to 0.23	1.02 ± 0.16	1.05 ± 0.12	0.825	0.24	− 0.48 to 0.95
Zone6	0.93 ± 0.15	0.98 ± 0.14	0.421	0.35	− 0.17 to 0.86	0.97 ± 0.15	1.01 ± 0.13	0.594	0.30	− 0.42 to 1.02
Zone7	0.82 ± 0.16	0.81 ± 0.24	0.979	− 0.05	− 0.56 to 0.46	0.84 ± 0.19	0.83 ± 0.15	0.948	− 0.06	− 0.78 to 0.65

CI, confidence interval

**Table 3:** Relation between stem anterior tilt alignment and BMD change at 24 months post-operatively in the Gruen zones

	Outlier versus non-outlier (anterior tilt)									
	HA full compaction					Tapered wedge				
	Outlier (*n* = 48)	Non-outlier (*n* = 11)	*p* value	Effect size (Cohen’s d)	95% CI	Outlier (*n* = 24)	Non-outlier (*n* = 32)	*p* value	Effect size (Cohen’s d)	95% CI
Zone1	0.91 ± 0.14	0.87 ± 0.15	0.261	− 0.28	− 0.94 to 0.38	0.88 ± 0.10	0.95 ± 0.13	0.055	0.59	0.05 to 1.13
Zone2	0.91 ± 0.12	0.89 ± 0.08	0.492	− 0.18	− 0.83 to 0.48	0.98 ± 0.13	1.00 ± 0.11	0.845	0.17	− 0.36 to 0.70
Zone3	1.01 ± 0.06	0.99 ± 0.03	0.249	− 0.36	− 1.02 to 0.30	1.02 ± 0.10	1.05 ± 0.08	0.532	0.34	− 0.20 to 0.87
Zone4	1.04 ± 0.06	1.03 ± 0.06	0.799	− 0.17	− 0.82 to 0.49	1.02 ± 0.05	1.01 ± 0.05	0.769	− 0.30	− 0.83 to 0.23
Zone5	1.04 ± 0.09	1.02 ± 0.04	0.638	− 0.24	− 0.90 to 0.42	1.06 ± 0.14	1.03 ± 0.11	0.434	− 0.24	− 0.77 to 0.29
Zone6	0.95 ± 0.15	0.98 ± 0.07	0.904	0.22	− 0.44 to 0.87	1.00 ± 0.13	1.01 ± 0.14	0.984	0.07	− 0.46 to 0.60
Zone7	0.81 ± 0.21	0.84 ± 0.13	0.638	0.15	− 0.50 to 0.81	0.84 ± 0.15	0.83 ± 0.16	0.784	− 0.06	− 0.59 to 0.47

CI, confidence interval

**Table 4:** Relation between stem absolute anteversion error and BMD change at 24 months post-operatively in the Gruen zones

	Outlier versus non-outlier (absolute anteversion error)									
	HA full compaction					Tapered wedge				
	Outlier (*n* = 24)	Non-outlier (*n* = 35)	*p* value	Effect size (Cohen’s d)	95% CI	Outlier (*n* = 28)	Non-outlier (*n* = 28)	*p* value	Effect size (Cohen’s d)	95% CI
Zone1	0.92 ± 0.11	0.90 ± 0.16	0.287	− 0.14	− 0.67 to 0.39	0.89 ± 0.11	0.96 ± 0.11	0.022*	0.64	0.10–1.17
Zone2	0.92 ± 0.12	0.90 ± 0.11	0.369	− 0.18	− 0.71 to 0.36	0.98 ± 0.10	1.00 ± 0.14	0.249	0.16	− 0.36 to 0.69
Zone3	1.01 ± 0.05	1.02 ± 0.06	0.937	0.18	− 0.34 to 0.70	1.03 ± 0.08	1.04 ± 0.10	0.571	0.11	− 0.41 to 0.64
Zone4	1.03 ± 0.04	1.05 ± 0.06	0.267	0.38	− 0.15 to 0.90	1.01 ± 0.05	1.02 ± 0.05	0.265	0.20	− 0.33 to 0.73
Zone5	1.01 ± 0.07	1.06 ± 0.09	0.105	0.61	0.08–1.14	1.04 ± 0.16	1.05 ± 0.14	0.653	0.07	− 0.46 to 0.59
Zone6	0.95 ± 0.15	0.96 ± 0.14	0.275	0.07	− 0.45 to 0.60	0.98 ± 0.11	1.04 ± 0.16	0.154	0.44	− 0.09 to 0.97
Zone7	0.82 ± 0.22	0.81 ± 0.19	0.930	− 0.05	− 0.57 to 0.47	0.79 ± 0.13	0.89 ± 0.17	0.048*	0.66	0.12–1.20

CI, confidence interval

*p < 0.05

**Table 5:** Comparison of stem alignment whether stem collar contacting to femoral calcar or not

	Stem collar contact to the femoral calcar (*N* = 31)	Stem collar does not contact to the femoral calcar (*N* = 28)	*p* value
Valgus	4.2 ± 2.0	1.9 ± 1.9	< 0.001
Anterior tilt	6.4 ± 1.9	5.5 ± 2.4	0.100
Absolute anteversion error	4.2 ± 4.6	6.3 ± 5.9	0.100

**Table 6:** Comparison of stem alignment among Door types of femoral bone shape

	Door type A (*n* = 4)	Dorr type B (*n* = 50)	Dorr type C (*n* = 5)	*p* value
Valgus	2.8 ± 2.0	2.8 ± 2.1	6.2 ± 1.7	0.004
Anterior tilt	6.3 ± 1.7	5.8 ± 2.3	7.6 ± 1.1	0.208
Absolute anteversion error	2.7 ± 2.9	5.5 ± 4.7	3.5 ± 6.8	0.396

Post-hoc power analysis was performed using G*Power [18]. For comparison between the groups, for a sample size of 59 versus 56 elements in the two groups, and a type-I error (α) of 0.05, the study is expected to provide a power (1-β) of 0.83 for detecting an effect size of 0.5. Comparing the outliers and non-outliers, we calculated the effect size by means and SDs based on the *Cohen’s d* for each parameter and 95% confidence interval for effect sizes (Tables 2, 3, 4) [19].

## Results

### Patients’ characteristics

Patients’ characteristics between the full HA compaction short stem and tapered-wedge stem groups was compared. The mean patient age at operation was 66.8 ± 11.1 years in the full HA compaction group, and 67.5 ± 9.1 years in the tapered-wedge group (*p* = 0.800). During clinical evaluation at 24 months postoperatively, the mean BMI was 22.8 ± 2.9 kg/m^2^ in the full HA compaction group and 23.7 ± 3.7 kg/m^2^ in the tapered-wedge group (*p* = 0.843). The JOA score was 96.1 ± 4.5 in the full HA compaction group and 95.9 ± 6.2 in the tapered-wedge group (*p* = 0.825). The UCLA score was 6.4 ± 1.6 in the full HA compaction group and 6.1 ± 1.5 in the tapered-wedge group (*p* = 0.268). The mean values for age, BMI, JOA score, and UCLA activity score were not significantly different between the groups.

### Stem insertion alignment was affected by stem design

Table 1 demonstrates that no significant differences in stem and canal anteversions were found between the HA compaction and tapered-wedge groups. The mean absolute value of surgical error (postoperative stem anteversion–canal anteversion) was 5.2° ± 4.8° (HA compaction) and 8.8° ± 9.1° (tapered-wedge), and the mean absolute value of anteversion error was significantly higher in the tapered-wedge group than in the HA compaction group (*p* = 0.039) (Table 1). The mean absolute values of valgus error were 3.1° ± 2.2° (HA compaction) and 1.8° ± 1.8° (tapered-wedge), and the error was significantly higher in the HA compaction group than in the tapered-wedge group (*p* < 0.001) (Table 1). The mean absolute values of anterior tilt error were 6.0° ± 2.2° (HA compaction) and 3.1° ± 2.1° (tapered-wedge), and the error was significantly higher in the HA compaction group than in the tapered-wedge group (*p* < 0.001) (Table 1).

### Peri-prosthetic BMD changes were similar between the groups

**Fig. 3:**
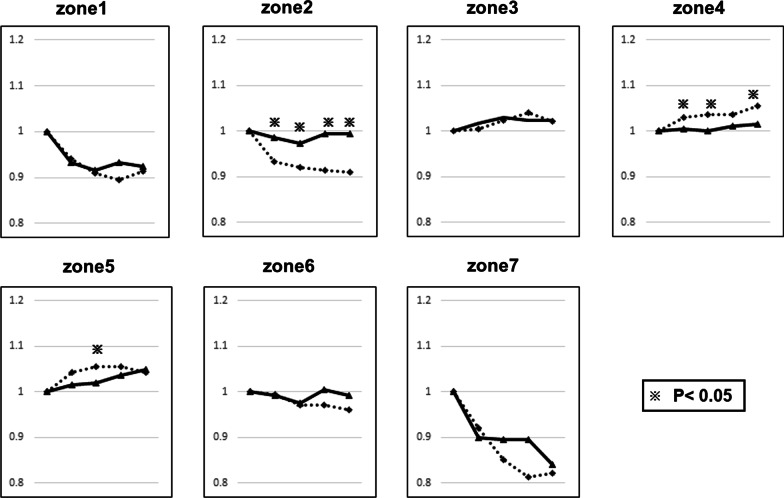
Bone mineral density (BMD) changes at 6, 12, 18, and 24 months postoperatively in the Gruen zone 7. Columns represent mean values of BMD changes. The dotted line indicates the value of HA compaction short stem. The solid line indicates the value of the short tapered-wedge stem. **※** Indicates *p* < 0.05 compared between two groups

Minimal BMD changes were found in the distal femur (Gruen zones 3, 4, and 5) and zone 6 in both stem types (Fig. 3). However, a significant BMD loss was observed in zones 1 and 7 at each timepoint in both groups compared with the BMD value obtained within 1 month postoperatively (full HA compaction: zone 1, 24 months, *p* < 0.001; zone 7, 24 months, *p* < 0.001) (tapered-wedge: zone 1, 24 months, *p* < 0.001; zone 7, 24 months, *p* < 0.001). On comparing the BMD changes between the groups, we found that the change in BMD of the tapered-wedge stem group was significantly higher in zone 2 at each timepoint. The change in BMD of the full HA compaction group was significantly higher in zone 4 (Fig. 3).

### Stem alignment affected peri-prosthetic BMD loss in tapered-wedge stem but not in HA compaction stem

Table 2 demonstrates that larger valgus alignment caused significant differences in peri-prosthetic BMD loss in Gruen zone 2 in the tapered-wedge group, but alignment did not affect BMD changes in the HA compaction group. Tables 2 and 3 demonstrate that peri-prosthetic BMD did not change between the outlier and non-outlier groups of anterior tilt in both HA compaction and tapered-wedge stems. Table 4 demonstrates that larger absolute anteversion errors caused significant differences in peri-prosthetic BMD loss in Gruen zones 1 and 7 in the tapered-wedge group. However, stem alignment of absolute anteversion error did not affect BMD changes in the HA compaction group (Table 4).

### HA compaction stem and tapered-wedge stem represent different patterns of contact to bone surface

**Fig. 4:**
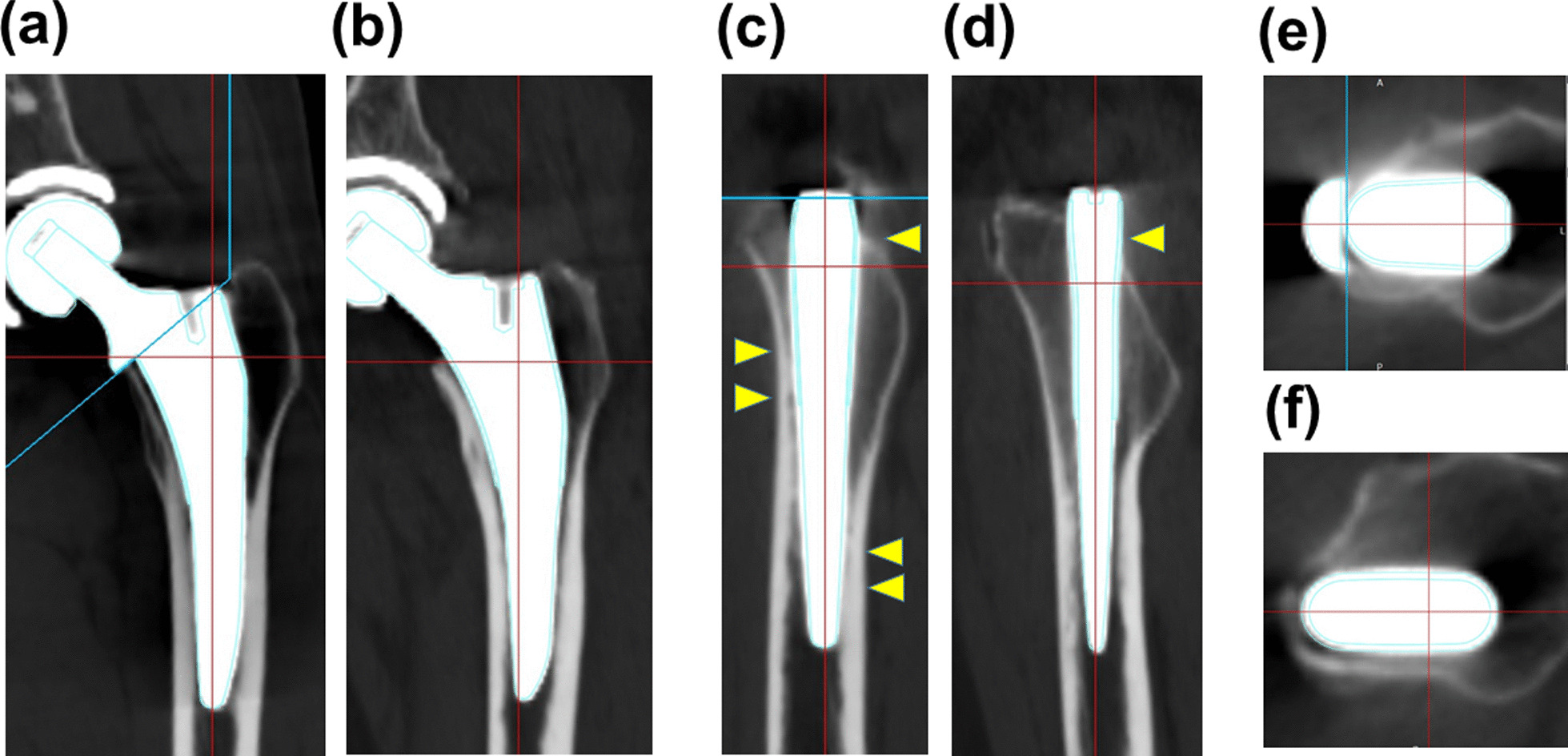
Three-dimensional reconstruction images of **a**, **c**, **e** the HA compaction short stem (ACTIS) and **b**, **d**, **f** the short tapered-wedge stem (Tri-Lock BPS). **a**, **b** Coronal view, **c**, **d** sagittal view. The yellow triangle indicates contact area of bone and stem **e**, **f** axial view

Figure 4a demonstrates the subject-specific differences of representative cases in HA compaction and tapered-wedge stems. In the HA compaction stem, the stem was in contact with the anterior wall, posterior edge of the proximal femur, and distal anterior wall in the sagittal view and widely occupied the antero-posterior area of the proximal femur in the axial view. Meanwhile, in the tapered-wedge stem, the stem contacts only the posterior edge of the proximal femur in the sagittal view and less occupied antero-posterior area of the proximal femur in the axial view (Fig. 4b).

### Relationship between stem collar contact and stem alignment

Table 5 demonstrates comparison of stem alignment, whether stem collar contacts the femoral calcar or not in the HA compaction stem. The mean value of stem valgus was significantly higher in the stem collar-calcar contact group than in the non-contact group (Table 5). The mean values of anterior tilt error and absolute value of anteversion error were not significantly changed between the stem collar-calcar contact and non-contact groups (Table 5).

### Relationship between femoral bone shape and stem alignment

Table 6 demonstrates comparison of stem alignments, according to the Dorr classification for femoral bone shape. The significant change in mean value of stem valgus error was noted among Dorr types (Table 6). A post-hoc analysis demonstrated that a significant mean value of valgus changes was found between Dorr types B and C (*p* = 0.003). The mean values of anterior tilt error and absolute value of anteversion error were not significantly changed among the Dorr types (Table 6).

## Discussion

In this study, we observed similar patterns of peri-prosthetic BMD changes in the full HA compaction short stem and tapered-wedge stem, although stem alignments of anteversion, valgus, and anterior tilt were different between the two groups. Furthermore, the stem alignment of the tapered-wedge stem affects peri-prosthetic BMD loss after THA, but that of the HA compaction stem did not.

Taniguchi et al. compared the stem insertion alignment of metaphyseal filling and short tapered-wedge stems and found that tapered-wedge stems had greater variation in the increase in anteversion than metaphyseal filling stems [20]. Our previous report demonstrated that the insertion alignment of short tapered-wedge stems was similar to that of straight stems during mini-invasive THA [21]. The current study demonstrated that the alignments of anteversion, valgus, and anterior tilt were different between HA compaction and tapered-wedge stems. This difference can be explained by stem geometry. The current study demonstrated that the HA compaction stem was in contact with the anterior wall, posterior edge of the proximal femur, and distal anterior wall in the sagittal view and widely occupied the antero-posterior area of the proximal femur in the axial view. The reason for contact pattern of HA compaction stem was explained by the stem profile that anterior–posterior width of HA-compaction stem was thicker than tapered-wedge stems, and the thicker anterior–posterior width of the HA-compaction stem may be fixed at larger anterior tilt and smaller anteversion errors along the femoral canal geometry. Another feature of the HA compaction short stem is the medial collar. In several cases, the medial collar may soon attach to the calcar region, and the stem alignment changes to valgus during bone compaction. We demonstrated that the mean value of stem valgus was significantly higher in the stem collar-calcar contact group than in the non-contact group, and the result can explain the reason for stem alignment changes to valgus during bone compaction in the stem collar-calcar contact group. However, we previously reported a relationship between the stem collar and periprosthetic BMD changes, and no differences was found in any of the Gruen zones between the stem collar-calcar contact and non-contact groups [5]. These findings supported our current result that stem alignment does not affect peri-prosthetic bone remodeling in the HA compaction stem.

Previous studies have investigated the influence of the proximal femoral canal shape on post-operative BMD changes in the femur [5, 22]. The pre-operative femoral canal’s morphology did not affect the BMD at two years post-operatively around the Zweymüller-type stem [22]. We previously reported that the proximal femoral canal’s shape affected post-operative BMD changes in the tapered-wedge stem, but did not in the full HA compaction stem group. The current study demonstrated that stem valgus error of HA compaction stem was higher in Dorr type C than in Dorr type B. These findings with our current result showed that stem alignment does not affect peri-prosthetic bone remodeling in the HA compaction stem.

Our current study showed findings similar to those of a previous report; excessive mismatch of stem and anatomical canal anteversions caused peri-prosthetic proximal BMD loss in short tapered-wedge stems [14]. However, stem insertion alignment did not affect the BMD changes in the HA compaction stem group. Kim et al. reported that the metaphyseal fitting stem, which was particularly characterized by metaphyseal fixation, predominantly indicated proximal load transfer and excellent peri-prosthetic BMD preservation in the proximal region of the stem [23]. The proximal profile of the HA compaction short stem represents a thicker antero-posterior width than the short tapered-wedge stem, and can be expected to be fixed in the proximal femoral cavity and provide rotational stability in addition to the effect of HA-coating for early bone remodeling.

The limitations of this study were as follows: First, this was not a randomized study but a retrospective cohort study. To evaluate the clinical and radiographic outcomes, an analysis of a randomized selection of patients is preferable. The backgrounds of the patients were similar in our study. Secondly, the sample size was small with respect to the evaluation of outcomes. In particular, the sample size for the comparison between outliers and non-outliers was underpowered for analysis. Finally, the preoperative peri-prosthetic BMD was not measured. We did not compare BMD values pre- and postoperatively.

## Conclusions

In our study, similar patterns of peri-prosthetic BMD changes were observed between the full HA compaction short stem and tapered-wedge stem. Stem insertion alignments of anteversion, valgus, and anterior tilt were different between the two types of stem groups because of the different stem geometries. Moreover, stem alignment of the tapered-wedge stem affects peri-prosthetic BMD loss after THA, but HA compaction stem did not due to wide occupation of the proximal femoral cavity.

## Data Availability

All data generated or analyzed during this study are included in this published article.

## Abbreviations

BMD: Bone mineral density
BMI: Body mass index
BPS: Bone preservation
CT: Computed tomography
DEXA: Dual-energy X-ray absorptiometry
HA: Hydroxyapatite-coated
JOA: Japanese Orthopedic Association
ROI: Regions of interest
SD: Standard deviation
THA: Total hip arthroplasty
UCLA: University of California Los Angeles
